# Selection of reliable reference genes for the normalisation of gene expression levels following time course LPS stimulation of murine bone marrow derived macrophages

**DOI:** 10.1186/s12865-017-0223-y

**Published:** 2017-10-03

**Authors:** Akane Tanaka, Joyce To, Bronwyn O’Brien, Sheila Donnelly, Maria Lund

**Affiliations:** 1 The School of Life Sciences, University of Technology Sydney, Ultimo, NSW Australia; 2 The Centre for Health Technologies, University of Technology Sydney, Ultimo, NSW Australia

**Keywords:** Macrophage, Lipopolysaccharide, Pro-inflammatory responses, RT-qPCR, Reference gene

## Abstract

**Background:**

Macrophages are key players in the initiation, perpetuation and regulation of both innate and adaptive immune responses. They largely perform these roles through modulation of the expression of genes, especially those encoding cytokines. Murine bone marrow derived macrophages (BMDMs) are commonly used as a model macrophage population for the study of immune responses to pro-inflammatory stimuli, notably lipopolysaccharide (LPS), which may be pertinent to the human situation. Evaluation of the temporal responses of LPS stimulated macrophages is widely conducted via the measurement of gene expression levels by RT-qPCR. While providing a robust and sensitive measure of gene expression levels, RT-qPCR relies on the normalisation of gene expression data to a stably expressed reference gene. Generally, a normalisation gene(s) is selected from a list of “traditional” reference genes without validation of expression stability under the specific experimental conditions of the study. In the absence of such validation, and given that many studies use only a single reference gene, the reliability of data is questionable.

**Results:**

The stability of expression levels of eight commonly used reference genes was assessed during the peak (6 h) and resolution (24 h) phases of the BMDM response to LPS. Further, this study identified two additional genes, which have not previously been described as reference genes, and the stability of their expression levels during the same phases of the inflammatory response were validated. Importantly, this study demonstrates that certain “traditional” reference genes are in fact regulated by LPS exposure, and, therefore, are not reliable candidates as their inclusion may compromise the accuracy of data interpretation. Testament to this, this study shows that the normalisation of gene expression data using an unstable reference gene greatly affects the experimental data obtained, and, therefore, the ultimate biological conclusions drawn.

**Conclusion:**

This study reaffirms the importance of validating reference gene stability for individual experimental conditions. Given that gene expression levels in LPS stimulated macrophages is routinely used to infer biological phenomena that are of relevance to human conditions, verification of reference gene expression stability is crucial.

**Electronic supplementary material:**

The online version of this article (doi:10.1186/s12865-017-0223-y) contains supplementary material, which is available to authorized users.

## Background

Macrophages play a significant role in the initiation and perpetuation of innate and adaptive immune responses. In this role, they perform multiple functions, including the uptake, processing and presentation of antigen, microbial killing, phagocytosis of apoptotic cells, and the secretion of cytokines, chemokines and chemical mediators [1]. The expression and secretion of immune modulators imparts the macrophage the ability to orchestrate immune responses, skewing them toward the pro-inflammatory, anti-inflammatory or regulatory arms of immunity. While pro-inflammatory responses are crucial to the elimination of pathogens, they are also central to the pathogenesis of autoimmune (for example type 1 diabetes, multiple sclerosis and rheumatoid arthritis) and pro-inflammatory (for example sepsis) diseases. Accordingly, much research has focused on understanding the responses of macrophages to pro-inflammatory conditions. These investigations often use murine bone marrow derived macrophages (BMDM), as a model mammalian macrophage system [2–4]. This is because BMDMs exhibit phenotypic and functional homogeneity and closely resemble ex vivo primary cells, thereby making them the preferable model, as opposed to other sources of primary macrophages or cell lines. Immortalised cell lines differ significantly in phenotype/function to primary murine macrophages. Primary resident macrophages of the peritoneum or lung are not naïve, and, therefore, differ phenotypically/functionally according to their previous immune experiences. Moreover, these cells are obtained in lower numbers, as compared to yields derived from bone marrow.

The pro-inflammatory response of macrophages is commonly investigated using lipopolysaccharide (LPS; a major component of bacterial membranes), as a biologically relevant inducer of inflammation [5–7]. The progression of this pro-inflammatory response in macrophages has been well characterised, with an initial induction phase of inflammation at 2 h following LPS exposure, a peak inflammatory phase at 6 h, and, finally, a resolution phase at 24 h post-stimulation [8–10]. The analysis of gene expression levels by RT-qPCR is often employed to study this pro-inflammatory response [11–13]. As a powerful tool, RT-qPCR is a rapid and sensitive technique with potential for high throughput, which allows the detection and quantitation of low abundance mRNA [14]. Despite these advantages, the accuracy and reliability of RT-qPCR data interpretation is dependent upon factors intrinsic to the preparation steps prior to RT-qPCR analysis, including RNA quality and quantity and reverse transcriptase (RT) efficiency. Indeed, it is crucial that the quantity of RNA input to the RT reaction be normalised. However, while necessary, this is not sufficient for direct comparisons of RT-qPCR data. A reference/normalisation gene(s), whose expression level is not regulated by the specific experimental conditions, is routinely included in RT-qPCR for all samples, thereby enabling normalisation of expression levels of the gene of interest (GOI) data [15, 16]. It is recommended that for each set of experimental conditions, the optimal reference gene(s) be determined [15]. However, in reality, few studies validate this important optimisation parameter. Rather, “traditional” reference genes, such as glyceraldehyde 3-phosphate dehydrogenase (*Gapdh*) or β-2 microglobulin (*B2m*), that are presumed to be stable in their expression levels, are generally selected as reference genes, despite the fact that they may not be expressed at consistent levels under the specific experimental conditions being studied. Normalisation of target gene expression levels to those of a reference gene which is, itself, regulated by the experimental conditions, likely affects measurements of comparative gene expression levels, thereby compromising data interpretation and biological conclusions made [17–19]. Thus, it is crucial that for any given experimental condition, one or more consistently expressed reference genes are identified and used [15].

To date, no optimal reference gene (or combination of reference genes) has been identified and validated for the normalisation of gene expression levels between control and LPS stimulated BMDMs over the time course of the pro-inflammatory response. Thus, the current study aimed to identify the most stably expressed reference genes during the peak (6 h) and resolution (24 h) phases of inflammation. A list of eight genes that have been used commonly for the normalisation of gene expression levels in BMDMs was initially investigated. From microarray analyses of untreated and LPS treated BMDMs at 6 h and 24 h post-LPS stimulation, three additional candidate reference genes, whose expression remained unchanged under these experimental conditions, were added to the list of commonly used reference genes. Thus, the expression levels of a total of 11 candidate reference genes were compared at each time point following LPS stimulation, and the optimal reference gene, or combination of reference genes, for each phase of the pro-inflammatory response was identified using NormFinder [20], GeNorm [21] and BestKeeper [22] softwares.

This study is the first to identify and validate *Hnrnpab* and *Stx5a*, as optimal reference genes for the normalisation of gene expression data during the peak and resolution phases, respectively, of the BMDM response to LPS. Moreover, this study demonstrates the consistency in expression levels of both *Hnrnpab* and *Stx5a* for peritoneal and RAW 264.7 macrophages stimulated with LPS. Importantly, the expression levels of these genes are more stable than those of *Gapdh* and *Actinb*, which are both commonly regarded as reference genes in the assessment of macrophage inflammatory responses. These observations demonstrate the importance of assessing the stability of reference genes for every experimental condition prior to normalisation of gene expression.

## Results

### The range of C_t_ values for candidate reference genes differs according to the time point post-LPS stimulation

**Fig. 1:**
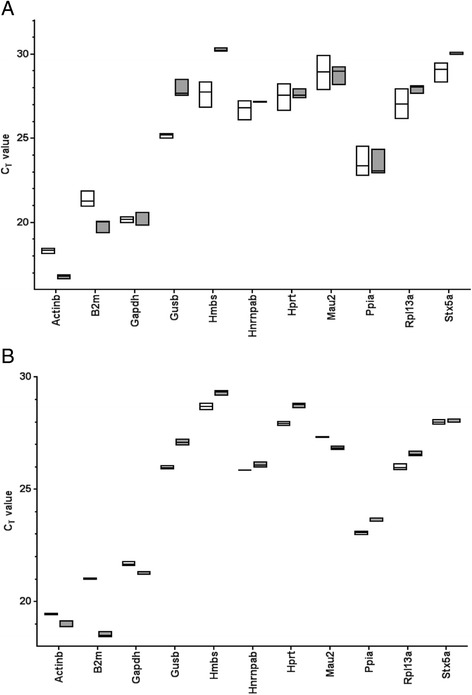
Ranges of C_t_ values of the 11 pre-selected reference genes in control and LPS stimulated BMDMs at 6 h and 24 h. C_t_ values were recorded for control and LPS stimulated (10 ng/ml) BMDMs at (**a**) 6 h and (**b**). 24 h. Plotted as boxes are the range of Ct values, with the included horizontal line identifying the mean, of triplicate biological replicates. The unfilled boxes represent control BMDMs, and the grey filled boxes represent LPS stimulated BMDMs

The aim of the current study was to identify the optimal reference gene, or combination of reference genes, for the normalisation of gene expression data in the commonly studied LPS stimulation model using BMDMs. We first identified a list of eight genes routinely used for the normalisation of gene expression data from experiments using BMDMs in general [23–25] or LPS stimulated BMDMs [26–35]. These candidate genes were *Actinb, B2m, Gapdh, Gusb, Hmbs, Hprt, Ppia* (cyclophilin A), and *Rpl13a*. We then identified candidate reference genes from a microarray data set comparing gene expression levels between control and LPS stimulated BMDMs at 6 h and 24 h post-exposure to LPS (Additional file 1: Table S1 and Additional file 1: Table S2, respectively). We selected three candidate reference genes whose expression remained unchanged between control and LPS-stimulated cells at both 6 h and 24 h, including chromatid cohesion factor homolog (*Mau2*; a gene involved in cell cycle), heterogenous nuclear ribonucleoprotein A/B (*Hnrnpab*; involved in mRNA processing) and Syntaxin 5a (*Stx5a*; a gene involved in autophagy; [36]). Thus, a total of eleven candidate reference genes were tested for the stability of their expression levels. The range of C_t_ values for untreated and LPS treated BMDMs at each time point, for each gene tested, are described in Fig. 1. The level of expression of genes differed, with some being highly expressed, for example, *Actinb* (mean (standard deviation [SD]): 6 h control; 18.34(0.18)), while others were expressed at much lower levels, for example, *Hmbs* (mean (SD): 6 h control; 27.73(0.81)).

The variance in expression levels of candidate reference genes, as indicated by the range of C_t_ values and SD, differed within a single treatment group. For example, within the control group at 6 h, the lowest variance in C_t_ values, as indicated by the lowest SD, was *Actinb* (SD = 0.18; Fig. 1a). The highest variance within the untreated group was for *Mau2* (SD = 1.06; Fig. 1a). Interestingly, in some cases, genes differed in their expression variability, dependent upon the time point following LPS stimulation. For example, in the control group, at 6 h, the variance in C_t_ values between replicates recorded for the gene *Rpl13a* was larger (range(SD) = 26.15–27.96(0.91)) than the variance observed for *Rpl13a* at 24 h post-LPS stimulation (range(SD) = 25.86–26.17(0.17)).

### The most stable reference gene differs according to the stage of inflammation

**Fig. 2:**
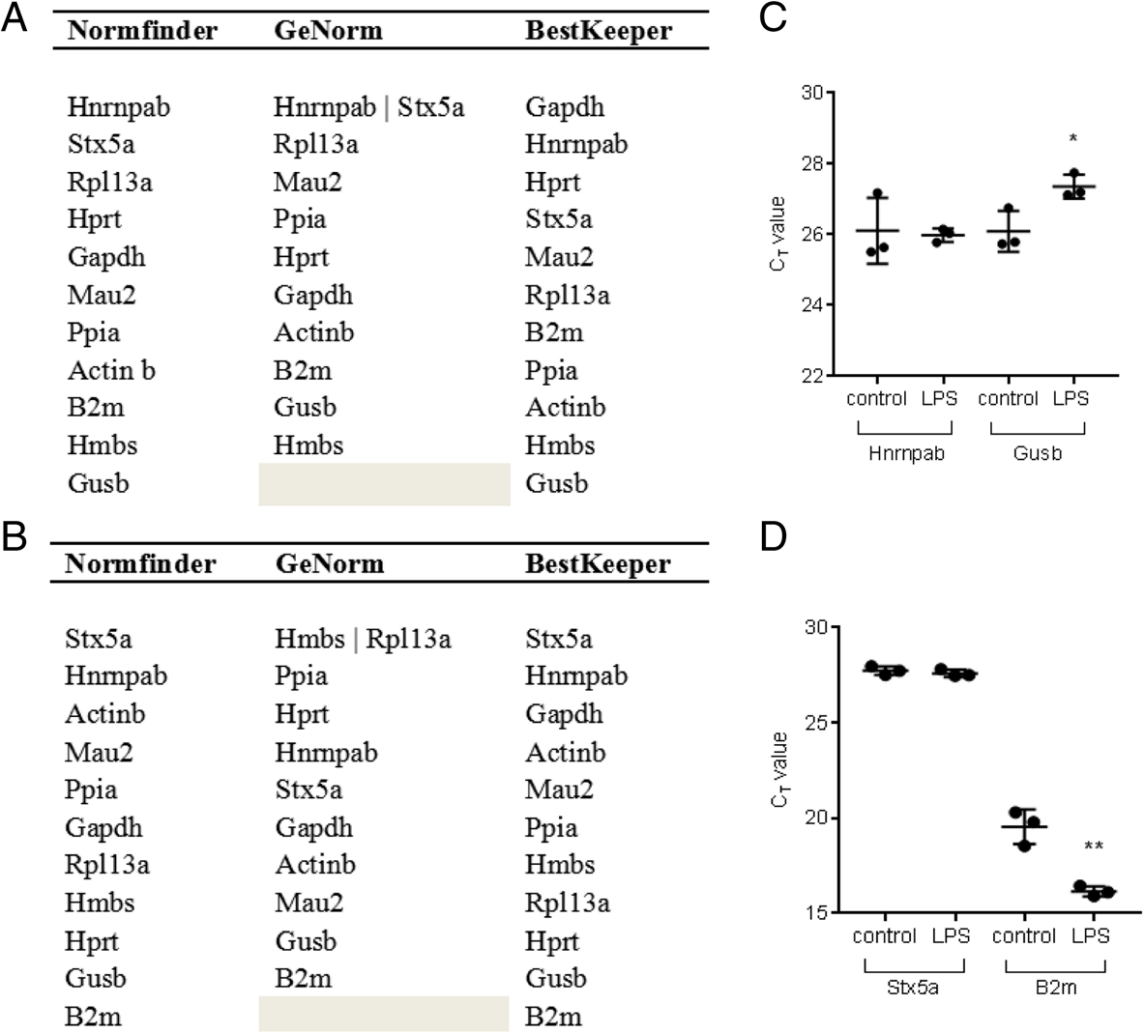
Stability ranking of reference genes in control and LPS stimulated BMDMs at 6 h and 24 h by Normfinder, GeNorm and BestKeeper softwares. C_t_ values were recorded for control and LPS stimulated (10 ng/ml) BMDMs at 6 h and 24 h. C_t_ values were transformed as instructed and applied to each reference gene analysis software. The tables above show the ranking of most to least stably expressed reference genes between control and LPS stimulated cells, from top to bottom, as identified by Normfinder, GeNorm and BestKeeper softwares, at (**a**) 6 h and (**b**). 24 h. The most and least stable reference genes were identified by NormFinder from an initial data set at 6 h and 24 h. The stability in expression levels of these selected genes were then analysed in an independent experiment by RT-qPCR and C_t_ values are shown at 6 h (**c**) and 24 h (**d**). The error bars represent means ± SD. The significance values were calculated by comparison of control and LPS treated samples as a group, at each time point. *p < 0.05, **p < 0.01

In order to identify the most consistently expressed reference gene candidate at each time point, the C_t_ values were inputted into GeNorm, NormFinder and BestKeeper programs. The analysis provided by NormFinder assigns a stability ranking to candidate reference genes using an algorithm which takes into account intra- and inter-variability (i.e. the variability in expression levels within and between the treatment groups, respectively) [20]. GeNorm determines the expression stability of a gene using a stepwise exclusion of the least stably expressed gene, generating an M value for each reference gene, and M values are then ranked. The Bestkeeper software assigns a ranking to each candidate reference gene based on the SD between samples. The rankings provided by each software for samples at 6 h and 24 h after LPS or vehicle exposure are described in Figs. 2a and b, respectively. There was broad agreement between the three programs regarding the two most and least consistently expressed reference genes between control and LPS stimulated groups at 6 h. At this time point, according to NormFinder and GeNorm, the most stable reference genes were *Hnrnpab* and *Stx5a*. Similarly, BestKeeper ranked *Hnrnpab* and *Stx5a* in the top four most stably expressed genes, at second and fourth, respectively. Application of all three softwares indicated that the two least stable genes were *Gusb* and *Hmbs*.

After 24 h LPS stimulation, there was less consensus among the softwares regarding the most reliable reference gene(s). NormFinder and BestKeeper were in agreement, ranking *Stx5a* and *Hnrnpab* as the two most stably expressed genes. GeNorm, however, identified *Hmbs* and *Rpl13a* as the most appropriate combination of genes for normalisation. In comparison, NormFinder and BestKeeper ranked *Hmbs* and *Rpl13a* seventh and eighth in stability, respectively. This discrepancy is likely due to the difference in the mathematical algorithms used to rank genes by NormFinder and BestKeeper, as compared to GeNorm. GeNorm assumes that two reference genes are not co-regulated. If they are co-regulated, they would score artificially high on the GeNorm ranking scale [37]. Indeed, the C_t_ values recorded for *Hmbs* and *Rpl13a* are both regulated in the same direction, in the LPS treated group, as compared to controls, that is, expression of each gene is lower in the LPS treated group, as compared to the controls, evident as a significantly higher C_t_ value (mean(SD); *Hmbs*: control: 28.7(0.2), LPS: 29.3(0.1), *p* = 0.0084; *Rpl13a*: control: 26.0(0.2), LPS: 26.6(0.1), *p* = 0.0078; Fig. 1b). The least stable reference genes identified consistently between the three softwares were *Gusb* and *B2m*.

To validate these findings, an independent experiment was performed, and the expression of the most and least stable reference genes, as identified by NormFinder software, was analysed by RT-qPCR. In agreement with the findings described above, there was no difference in C_t_ values observed for *Hnrnpab*, between the control and LPS treated samples, at 6 h (Fig. 2c). In the above-mentioned analysis, *Gusb* was identified as the most unstable reference gene under these particular experimental conditions, and was clearly regulated by LPS exposure at 6 h. In agreement with these findings, the C_t_ values observed for *Gusb* were significantly higher following LPS exposure at 6 h (*p* = 0.0304), indicating that LPS down-regulated *Gusb* gene expression levels. After 24 h exposure to LPS, *Stx5a* was identified as the most stably expressed gene between control and treated cells. This was validated in the independent experiment, with no difference seen in C_t_ values between the two treatments (Fig. 2d). In agreement with the above-mentioned analysis, C_t_ values observed for *B2m* were significantly lower in the LPS treated BMDMs, as compared to controls (*p* = 0.0034), indicating that LPS up-regulated *B2m* expression at this time point.

**Fig. 3:**
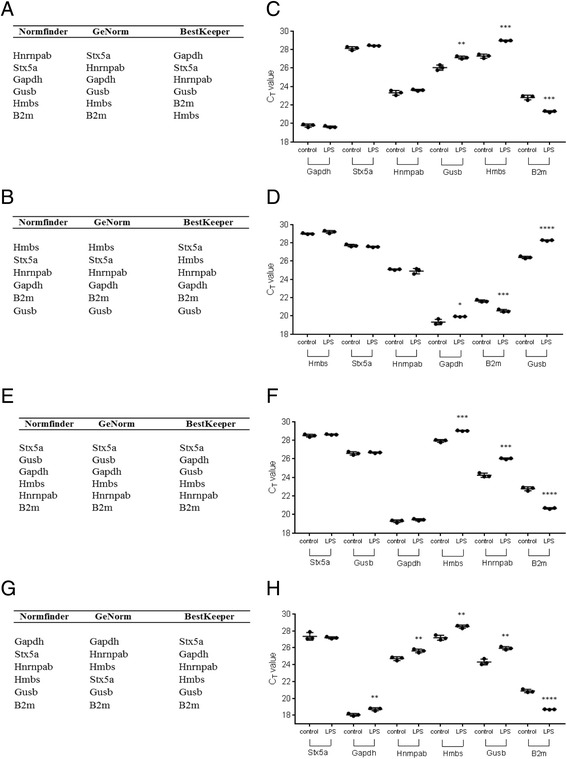
Stability ranking of select reference genes in LPS treated RAW 264.7 cells and peritoneal macrophages at 6 h and 24 h. C_t_ values were recorded for control and LPS (10 ng/ml) stimulated RAW 264.7 cells and peritoneal macrophages at 6 h (**a-d**) and 24 h (**e-h**). C_t_ values were transformed as instructed and applied to the 3 reference gene analysis softwares. The tables above show the ranking of most to least stably expressed reference genes between control and LPS stimulated cells in (**a, e**). RAW264.7 cells and (**b, g**) peritoneal macrophages, from top to bottom, as identified by Normfinder, GeNorm and BestKeeper softwares. The C_t_ values are plotted for LPS stimulated (**c, f**). RAW 264.7 cells and (**d, h**) peritoneal macrophages. The error bars represent means ± SDs. The significance values were calculated by comparison of control and treated samples as a group, at each time point. *p < 0.05, **p < 0.01, ***p < 0.001, ****p < 0.0001

To investigate the applicability of both *Hnrnpab* and *Stx5a* as reference genes in differently sourced macrophages, we compared the expression levels of six of the 11 candidate genes (three of the most stable and three of the least stable) in murine peritoneal macrophages and in the RAW 264.7 murine macrophage cell line. For all genes the C_t_ values were recorded for control and LPS stimulated (10 ng/ml) cells at 6 h (Figs. 3a-d) and 24 h (Figs. 3e-h). Consistent with the stability of genes in LPS stimulated BMDMs, there was no difference in C_t_ values observed for *Hnrnpab*, between the control and LPS treated cohorts, at 6 h. In addition, *Stx5a* was again identified as the most stably expressed gene between control and treated cells 24 h after LPS treatment, with no difference seen in C_t_ values between the two treatments. This additional study therefore validates *Hnrnpab* and *Stx5a* as suitable reference genes in these multiple sources of macrophages and at multiple time points.

### The choice of reference genes for data normalisation can affect biological conclusions

**Fig. 4:**
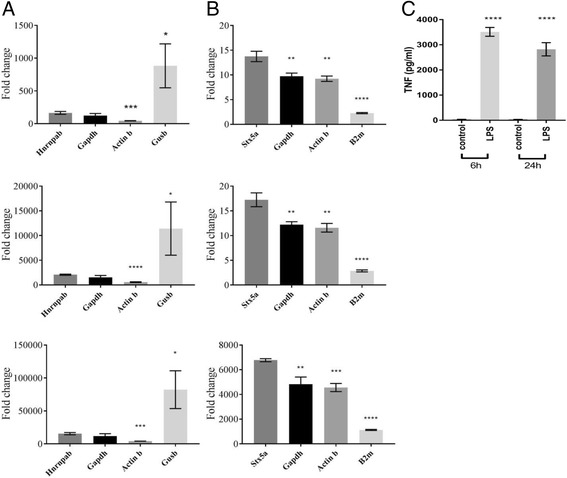
Fold change in TNF, IL-1β and NOS2 gene expression levels at 6 h and 24 h as calculated using different reference genes. Fold change in expression of TNF (top row), IL-1β (middle row) and NOS2 (bottom row) in the LPS treated group, as compared to the control group, at (**a**) 6 h and (**b**). 24 h, was calculated using the ΔΔC_t_ method, as described in the methods section. For normalisation, the most (*Hnrnpap, Stx5a*) and least consistently expressed reference genes (*Gusb, B2m*), as identified by Normfinder software analysis, and the traditional choice of *Actinb* and *Gapdh* were used for the calculation of ΔC_t_. (**c**). The release of TNF from control and LPS stimulated BMDM was measured by ELISA at 6 h and 24 h of LPS exposure. Bars represent the mean ± SD. *p ≤ 0.05, **p ≤ 0.01, ***p ≤ 0.001, ****p ≤ 0.0001

To demonstrate the impact and importance of selecting the most stable reference gene(s) for data normalisation, we analysed the relative expression of three genes whose increased expression levels characterise the pro-inflammatory response in BMDMs. The expression of IL-1β and TNF (both pro-inflammatory cytokines) and NOS2 (inducible nitric oxide synthase, an enzyme produced during the pro-inflammatory response) were assessed by RT-qPCR, and then normalised using the most and least stable reference genes, as identified by NormFinder software. At 6 h post-LPS stimulation, the fold change in expression levels of TNF was significantly higher in the LPS treatment group when gene expression data was normalised to the least stable reference gene, *Gusb* (mean ± SD: 883 ± 193), as compared to when normalisation was performed using the most stable reference gene, *Hnrnpab* (mean ± SD: 165 ± 12; *p* = 0.0208; Fig. 4a). When normalised to the traditional choice of housekeeping gene, *Gapdh*, the fold change in expression of TNF was similar (mean ± SD: 123 ± 19) to that seen when *Hnrnpab* was used for normalisation, which is consistent with its top stability ranking by BestKeeper software analysis. In contrast, using *Actinb*, which was ranked poorly by all 3 analytical programs, reduced the change in TNF gene expression by 3-fold (mean ± SD: 44 ± 2). Similarly, following 24 h exposure to LPS, the fold change in expression levels of TNF in the LPS-treated group was significantly different when normalised to the most inconsistent reference gene, *B2m* (mean ± SD: 2 ± 0), as compared to normalisation using the most consistent reference gene, *Stx5a* (mean ± SD: 13 ± 1; *p* < 0.0001; Fig. 4b). At this 24 h time point, *Gapdh* and *Actinb* were both ranked midway between *B2m* and *Stx5a* with respect to stability, and in agreement with this finding, when they were used as reference genes the fold change in expression of TNF was significantly less than that obtained using *Stx5a* as the reference gene (mean ± SD: 9.7 ± 0.4 and 9.2 ± 0.3). Similarly, normalisation using an inconsistently expressed reference gene also altered the measurements of fold change in gene expression for IL-1β and NOS2 at each time point (Figs. 4a, b).

Collectively, this data clearly demonstrates that normalisation of gene expression data using an inconsistently expressed reference gene can alter the magnitude of fold changes in expression, which may impact upon the interpretation of the biological significance of the study. The two-fold change in gene expression of TNF observed at 24 h after LPS stimulation when data was normalised to *B2m* expression levels may be interpreted as not a biologically significant increase above control. However, when data was normalised to *Stx5a*, there was a significant increase in TNF gene expression levels above control. Importantly, this increase in expression at the mRNA level was corroborated at the protein level as amounts of secreted TNF at 24 h post-LPS stimulation were significantly higher than control samples (mean ± SD: 2821 ± 152 pg/ml, as compared to 34 ± 1 pg/ml, respectively; *p* < 0.0001; Fig. 4c). Consequently, in this example, normalisation to an inconsistent reference gene resulted in an underestimation of the magnitude of the pro-inflammatory response. These data demonstrate that the selection of consistently expressed reference genes, under the experimental parameters chosen, is crucial for the accurate interpretation of data, and, ultimately, to the elucidation of biological responses.

## Discussion

Murine BMDMs are well established as a model primary macrophage population for the study of the inflammatory response. Commonly, LPS is used as a pro-inflammatory stimulus to induce gene expression in BMDMs, to investigate this pro-inflammatory response. Given the efficiency, sensitivity and robustness of RT-qPCR, this technique is commonly employed to measure gene expression levels in the study of the macrophage inflammatory response. The use of RT-qPCR necessitates normalisation of gene expression data to a reference gene, which is stably expressed under the experimental conditions used. In fact, normalisation of GOI data to a reference gene whose expression is regulated by the experimental condition can lead to erroneous results, and therefore incorrect conclusions [17–19]. Thus, the current study aimed to identify the optimal reference genes for normalisation of gene expression data for experiments using BMDMs stimulated with LPS over the course of the inflammatory response, specifically the peak (6 h) and resolution (24 h) phases. The data presented herein demonstrates that the expression levels of “traditional” reference genes may vary significantly in this cell model under pro-inflammatory conditions. Moreover, we demonstrate that the normalisation of GOI data using genes that are regulated by the experimental conditions can have a significant impact on the interpretation of RT-qPCR data and, ultimately, the validity of conclusions drawn.

A review of the literature identified eight candidate reference genes that have been used for the normalisation of gene expression data for BMDMs. We set out to evaluate the stability of these genes, and to identify any additional reliable reference genes. Microarray analyses of LPS stimulated BMDMs at 6 h and 24 h identified three candidate reference genes, whose expression remained unchanged between control and LPS treated cells. Two of the reference genes identified from the microarray analysis, *Hnrnpab* and *Stx5a*, were ranked as the most stable reference genes for normalisation of gene expression data, at both 6 h and 24 h. It should be noted that several commonly used reference genes, although not ranked in first or second position, were ranked in the top five for stability, and would, therefore, still be considered suitable for the normalisation of gene expression data. For example, at 6 h, *Rpl13a, Hprt* and *Gapdh* ranked third, fourth and fifth, respectively, according to NormFinder analysis. At 24 h, *Actinb* and *Ppia* were ranked third and fifth, respectively. Importantly, the current study identified several genes that should be excluded as reference genes under these particular experimental conditions, including *Hmbs* and *Gusb* after 6 h of BMDM exposure to LPS, and *Gusb* and *B2m* after 24 h LPS exposure. These genes were ranked lowest for stability by all three programs, and, therefore, their expression levels were clearly regulated by LPS exposure. The current study also indicates that *B2m* and *Actinb* are unsuitable candidates for normalisation of expression data at 6 h post-LPS stimulation, as their expression levels are modulated by LPS exposure, and, accordingly, these genes were ranked in the lowest five for stability by all three programs.

Despite the publication of the MIQE (Minimum Information for Publication of Quantitative. Real-Time PCR Experiments) guidelines [15], incorrect presumptions that “traditional” reference genes are stably expressed, without reported validation under specific experimental conditions, are widespread among gene expression studies [38]. The stability of traditional references genes, such as *Gapdh* and *Actinb*, has previously been called into question [39–41]. In agreement, the current study further highlights the pitfalls of using such an approach for the selection of reference genes for data normalisation. The commonly used reference gene, *Actinb*, although stably expressed at 24 h post-LPS stimulation (ranked third and fourth, by NormFinder and BestKeeper, respectively), was found to be one of the most highly regulated genes when cells were exposed to LPS for 6 h (ranked in the lowest four by all three programs). Similarly, levels of *Hprt* gene expression at 6 h post LPS-treatment were stable, however, at 24 h, expression levels were down-regulated by exposure to LPS. These results demonstrate that reference gene expression levels are modulated by their cellular conditions, and highlight the requirement that all genes must be tested for expression stability under the specific experimental conditions being studied.

The impact of normalising expression data using unstable reference genes has been demonstrated in several previous publications [17–19]. The current study demonstrates the importance of normalisation using a reference gene that is stably expressed under the experimental conditions of inducing a pro-inflammatory response over time. The data demonstrated that normalisation using a reference gene that was not stably expressed falsely diminished the magnitude of the inflammatory response measured. In fact, the relative level of TNF gene expression (~2 fold increase) calculated by normalisation to an inappropriate reference gene 24 h after LPS treatment indicated an almost complete absence of the pro-inflammatory response to LPS. This result was in stark contrast to the actual higher levels of secreted TNF, and TNF at the level of gene expression when data was normalised to a stable reference gene, following LPS exposure at 24 h as compared to controls. Gene expression of TNF is highly regulated by LPS exposure (~13-fold increase at 24 h). Thus, caution must be exercised when normalising gene expression data for a GOI that is less highly regulated (i.e. a gene that exhibits smaller changes in expression levels attributed to the experimental conditions). Smaller changes in gene expression would be more likely to be masked by normalisation to an inappropriate reference gene.

This study has demonstrated that it is crucial to determine the stability of reference genes under the specific experimental conditions employed. This is perhaps of paramount importance when studying the responses of cell types, such as macrophages, which are highly responsive to even subtle changes in the inflammatory milieu of their environment. We suggest a potential approach for the identification of stable reference genes for studying new experimental conditions. Initially, a review of the literature should be undertaken to identify several reference gene candidates. Next, if published array data is available, this can be used to identify genes whose expression levels remain unchanged under experimental conditions. Next, the stability of several candidate reference genes should be determined under experimental conditions by RT-qPCR. Finally, two or more programs should be used to identify the most stable reference gene. However helpful these programs may be in identifying the most stably expressed gene, it is still important to confirm the stability of the chosen genes by examining C_t_ values. For example, we have shown that, at 24 h, GeNorm identified a combination of genes as most stable, that were in fact regulated by LPS exposure, presumably because the algorithm used by GeNorm to rank stability assumes there is not co-regulation of the candidate reference genes.

## Conclusions

The current study identified the most stably expressed genes for use in the normalisation of gene expression data during the course of the pro-inflammatory response of primary murine macrophages to LPS. The two most stably expressed genes were identified from microarray analyses of BMDMs exposed to LPS, and, here, make their debut as reference genes. The data presented also confirms the stability of other reference genes used previously in the literature for normalisation of gene expression data in this inflammatory BMDM model. Importantly, we have identified several “traditional” reference genes that are often assumed to be stable, whose expression levels are, in fact, strongly regulated during the BMDM inflammatory response to LPS. We recommend that these genes not be used for the normalisation of gene expression data in this model, due to the likelihood of erroneous results, and the potential for invalid experimental conclusions to be drawn. This also highlights the need for experimental validation of reference gene stability under specific experimental conditions, and we have outlined a potential approach for identifying stable reference genes.

## Methods

### Differentiation and stimulation of macrophages

Bone marrow derived macrophages (BMDMs) were differentiated from the bone marrow of 6–8 week old female Balb/c mice (ARC, Perth, Australia), as previously described [42]. All procedures were in accordance with the guidelines of the UTS Animal Care and Ethics Committee (Protocol number: 2012–080). Briefly, bone marrow was flushed from freshly isolated femurs and tibias with RPMI 1640 (Life Technologies). Cells were collected by centrifugation (300×g, 5 min), and resuspended at a density of 2 × 10^6^ cells/ml in BMDM media (RPM1 1640, supplemented with 10% *v*/v heat inactivated, certified low endotoxin, foetal calf serum (Life Technologies, catalogue number 10082147), beta-mercaptoethanol (715 nM), penicillin/streptomycin (1% *v*/v, Life Technologies) and macrophage colony-stimulating factor (M-CSF, 50 ng/ml, eBioscience). Cells (2 × 10^7^ cells in 10 ml media) were then plated into sterile 90 mm petri dishes (Techno Plas, catalogue number S90001G). Cells were differentiated for 6 days, with the addition of 10 ml media at Day 3. At Day 6, media and non-adherent cells were removed, remaining non-adherent cells were rinsed away with 10 ml sterile saline (Baxter Healthcare), and then 10 ml fresh BMDM media was added. Cells were detached by gently scraping into media and collected by centrifugation (300×g, 5 min). The purity of the BMDM population was determined at day 6 by staining for CD11b by flow cytometry. The purity of the BMDM population was always greater than 98% CD11b^+^ (data not shown).

Peritoneal lavages were collected from 6 to 8 week old female Balb/c mice. Macrophages were isolated from peritoneal exudate cells (PECs) by adherence to plastic in serum-free media.

BMDMs, peritoneal and RAW264.7 macrophages, were seeded at 2 × 10^6^ cells in 2 ml of media in 6 well tissue culture plates and allowed to adhere for 1.5 h before stimulation with LPS (from *E. coli*, 0111.B4; Sigma Aldrich) at a concentration of 10 ng/ml. Cells were incubated with LPS for 6 h or 24 h at 37 °C/5%CO_2_.

### Isolation of RNA

Supernatants were removed from cell samples, and wells were rinsed twice with sterile saline. RNA was isolated from cell samples using the Isolate II RNA mini kit (Bioline, Australia). Genomic DNA (gDNA) was removed by treatment with DNase I (Sigma Aldrich), according to the manufacturer’s instructions. The absence of contaminating gDNA was demonstrated by the absence of a product in wells using “no RT” control samples, which included all components of the cDNA synthesis reaction, except reverse transcriptase. The quality of RNA was assessed by spectrophotometry (NanoDrop One/One^c^, Thermofisher Scientific). Ratios for all samples are reported in Additional file 2 Table S3.

### cDNA synthesis

For the synthesis of cDNA, 500 ng RNA was used with the Superscript First Strand Synthesis Kit (Thermofisher Scientific), primed with random hexamers, according to the manufacturer’s instructions. The resultant cDNA was stored at −20 °C until RT-qPCR, when it was diluted 1/10 in RNAse/DNAse free water.

### RT-qPCR

RT-qPCR was performed using the QuantStudioFlex 12 K instrument (Applied Biosystems). Taqman gene expression assays listed in Table 1 were used according to the manufacturer’s instructions, with cDNA (5 ng) in technical triplicates. The PCR program was as follows: UNG activation (50 °C, 2 min), UNG inactivation (95 °C, 10 min), followed by 40 cycles of denaturation (95 °C, 15 s) and annealing/extension (60 °C, 1 min). The mean C_t_ values were calculated from technical triplicates. For the calculation of fold change in expression, the ΔΔC_t_ method was used [43]. For samples with C_t_ values that were greater than 35 was considered a negative, however for the purpose of calculating a fold change, these samples were assigned a C_t_ value of 35. The mean ΔC_t_ value of the control (untreated) samples was calculated, and individual ΔC_t_ values were calculated for experimental replicates within the LPS treated samples, using the formula: ΔC_t_ = C_t_ target gene – C_t_ reference gene. The ΔΔC_t_ value for each LPS treated sample was calculated as the ΔC_t_ LPS sample – mean ΔC_t_ control samples. This was then transformed into a fold change value for each LPS sample using the calculation 2^-ΔΔCt^.

**Table 1:** Taqman gene expression assays

Gene		Assay ID	Dye
Actinb	β-actin	Mm00607939 sl	FAM-MGB
B2m	β-2-microglobulin	Mm0043762 ml	FAM-MGB
Gapdh	Glyceraldehyde-3-phosphate dehydrogenase	Mm99999915 gl	FAM-MGB
Gusb	β-glucuronidase	Mm01197698 ml	FAM-MGB
Hmbs	Hydroxymethylbilane synthase	Mm01143545 ml	FAM-MGB
Hnrnpab	Heterogeneous Nuclear Ribonucleoprotein A/B	Mm1288699 ml	FAM-MGB
Hprt	Hypoxanthine-guanine phosphoribosyltransferase	Mm00446968_ml	FAM-MGB
Mau2	MAU2 Sister Chromaid Cohesion Factor	Mm00512415 ml	FAM-MGB
Ppia	Peptidylprolyl Isomerase A	Mm02342430 gl	FAM-MGB
Rpl13a	Ribosomal protein L13a	Mm01612987 gl	FAM-MGB
Stx5a	Syntaxin 5a	Mm00502335 ml	FAM-MGB

### Gene expression microarray analysis

BMDMs were treated with vehicle or LPS, for 6 h and 24 h, and then RNA was isolated using Trizol reagent (Life Technologies) and the Qiagen Rneasy Plus Mini Kit (Qiagen). The aqueous phase of the Trizol preparation was obtained, according to the manufacturer’s instructions, and was applied to the gDNA-eliminating column of the RNeasy Plus Mini Kit, and then RNA was isolated according to the instructions of the manufacturer (Qiagen). RNA was submitted to the Ramaciotti Centre for Genomics (UNSW, Australia) for gene expression microarray analyses using the Affymetrix Mouse Gene 2.1ST array. Array data were analysed using Partek Genomics Suite (Partek Inc. USA). Data files were grouped according to treatment (control or LPS) and individual gene lists for 6h and 24h LPS stimulation were generated by one way ANOVA comparison of the two groups, with a fold change cut off of −1.<fold change<1.4 (Additional file 1 Tables S2 and S3, respectively). Then, the two lists (6h and 24h) were combined, and a list of genes that were common to both lists was generated, and three candidate reference genes were then selected.

### Measurement of TNF in supernatants

Levels of secreted TNF in the supernatants were quantified by ELISA (BD Pharmingen), according to the manufacturer’s instructions.

### Statistical analyses

The softwares GeNorm [21], NormFinder [20] and BestKeeper [22] were used according to the instructions supplied. This required that raw C_t_ values were transformed to different input formats for GeNorm and NormFinder analyses. For analysis using BestKeeper software, raw C_t_ values were inputted. For the statistical comparison of two groups, an unpaired, two-tailed t test was used in GraphPad Prism 7 software (GraphPad). *P* values indicated in the figure legends.

## Additional files

Additional file 1: **Tables S1 and S2.** Gene expression in murine macrophages treated with LPS**.** Microarray analysis of gene expression in macrophages treated with LPS for 6 h (**Table S1.**) and 24 h (**Table S2.**) (XLSX 1623 kb)

Additional file 2: **Table S3.** Assessment of RNA quality. Spectrophotometry analysis of the quality of RNA used in all qRT-PCRs. (DOCX 17 kb)

## Abbreviations

BMDM: Bone marrow derived macrophages
GOI: Gene of interest
LPS: Lipopolysaccharide
MIQE: Minimum information for publication of quantitative real-time PCR experiments
NOS2: Nitric oxide synthase
PEC: Peritoneal exudate cells
RT: Reverse transcriptase
SD: Standard deviation
