# Supplementary material for: Selection of reliable reference genes for the normalisation of gene expression levels following time course LPS stimulation of murine bone marrow derived macrophages
Source: BMC Immunol. 2017 Oct 3;18:43. doi: 10.1186/s12865-017-0223-y (PMC5627409; doi:10.1186/s12865-017-0223-y)
Supplement: Supplementary file 2 — Table S3. Assessment of RNA quality. Spectrophotometry analysis of the quality of RNA used in all qRT-PCRs. (DOCX 17 kb) [file 12865_2017_223_MOESM2_ESM.docx]

**Supplementary Table 3.** 260:280 and 260:230 ratios for assessment of RNA quality.

| **Time (h)** | **260:230** | | | | | | **260:280** | | | | | |
| --- | --- | --- | --- | --- | --- | --- | --- | --- | --- | --- | --- | --- |
|  | Control | | | LPS | | | Control | | | LPS | | |
|  | A | B | C | A | B | C | A | B | C | A | B | C |
| 6 | 1.96 | 2 | 2.14 | 2.14 | 2.16 | 2.1 | 2.19 | 2.17 | 2.2 | 2.18 | 2.16 | 2.22 |
| 24 | 1.78 | 2.03 | 2.08 | 1.91 | 2.18 | 2.02 | 2.17 | 2.26 | 2.18 | 2.18 | 2.18 | 2.18 |
